# Effect of a lay counselor delivered integrated maternal mental health and early childhood development group-based intervention in Siaya County, Kenya: A quasi-experimental longitudinal study

**DOI:** 10.1016/j.jad.2021.06.002

**Published:** 2021-06-13

**Authors:** Eunsoo Timothy Kim, Tobias Opiyo, Pauline S. Acayo, Margaret Lillie, John Gallis, Yunji Zhou, Michael Ochieng, Samwel Okuro, John Hembling, Elena McEwan, Joy Noel Baumgartner

**Affiliations:** a Duke Global Health Institute, Durham, NC, USA; b Catholic Relief Services, Nairobi, Kenya; c Department of Biostatistics & Bioinformatics, Duke University, Durham, NC, USA; d B&M Consult, Nairobi, Kenya; e Catholic Relief Services, Baltimore, MD, USA; f School of Social Work, University of North Carolina at Chapel Hill, Chapel Hill, NC, USA

**Keywords:** Mental health, Depression, Early stimulation, Early child development, Cognitive behavior therapy, Lay counselor, Task-shifting, Sub-Saharan Africa

## Abstract

**Background:**

Maternal mental health is linked to early childhood development; yet there is a gap in evidence-based interventions for low-resource settings. This study estimates the impact of ‘Integrated Mothers and Babies Course and Early Childhood Development’ (iMBC/ECD), a cognitive-behavioral, group-based intervention, on maternal depression and early childhood social-emotional development in Siaya County, Kenya.

**Methods:**

This quasi-experimental study enrolled 417 pregnant women and mothers of children under age 2 across two sub-counties in Siaya County. The intervention area had 193 women in 23 groups implementing iMBC/ECD and the control area had 224 women in 30 groups exposed to ECD only content. Mother/index child dyads were followed for two years. To estimate the causal treatment effect from the non-randomized design, we implemented the propensity score weighting method with inverse probability weights.

**Results:**

At baseline, 10.2% of participants endorsed moderate/severe depressive symptoms. At 14-months post-intervention, 7.4% endorsed moderate/severe depression. Overall, iMBC/ECD intervention did not have a significant impact on reducing maternal depression or improving children’s social and emotional development. However, sub-group analyses revealed that iMBC/ECD was associated with lowered depressive symptoms among women with no/low education, four or more children and/or no experience of intimate partner violence in the past year. Women with high program attendance (more than half of 14 sessions) also experienced consistently fewer depressive symptoms compared to those with lower attendance.

**Limitations:**

Non-randomized study, sub-group analyses are exploratory.

**Conclusions:**

The iMBC/ECD program may have the potential to improve maternal mental health and early child development for more targeted vulnerable populations.

## 1. Introduction

Improving maternal mental health is an important public health priority in low- and middle-income countries (LMICs) given women’s elevated risk of depression during the perinatal period. In LMICs, the average prevalence of depression is about 16% in the antenatal period and around 20%i in the postnatal period (Fisher et al., 2012). The mounting toll of poor maternal mental health is not only detrimental to the mothers’ own health and safety but on children’s developmental outcomes as well (Herba et al., 2016; Wachs et al., 2009; WHO, 2020a). A review examining the associations between maternal depression and child outcomes found that depression during pregnancy and in the postnatal period can have adverse effects on children’s behavioral and developmental outcomes (Herba et al., 2016). Specifically in the postnatal period, maternal depression can adversely affect children’s cognitive, social and emotional development indirectly through altered interactions between the mother and her child (Herba et al., 2016).

In Sub-Saharan Africa, 66% of under-5 children are at risk of not reaching their full developmental potential which includes cognitive, socio-emotional, behavioral and educational outcomes (Black et al., 2017). This is especially concerning as experiences in the early period of life are known to be instrumental in determining later life health outcomes and income earning potential (Black et al., 2017; Chan et al., 2016; Daelmans et al., 2016; Machel, 2016). Hence, addressing the dual burden of maternal depression and suboptimal children’s development should be prioritized in this context. The Nurturing Care Framework and the recent World Health Organization (WHO) guidelines on improving early childhood development (ECD) also recognize the critical role of maternal mental health for ECD and recommend that interventions integrate support for caregiver mental health with early childhood health and development services (WHO, 2020b).

The Government of Kenya has endorsed the Nurturing Care Framework and it is now included in a national policy document for community health (Kenya Ministry of Health, 2020). In Western Kenya, Siaya County has a county-wide ECD strategy in place that includes prioritizing evidence-based programming (Gray et al., n.d.). However, there is still a dearth of information regarding early learning opportunities and responsive caregiving practices for Kenya (UNICEF and Countdown to 2030: Women’s, 2019). The evidence base for effective, integrated maternal mental health and ECD interventions in low-resource settings is limited as well (Maselko et al., 2015; Singla et al., 2015; Turner et al., 2016).

An intervention called the *Integrated Mothers and Babies Course & Early Childhood Development* (iMBC/ECD) program was implemented by lay counselors in Siaya County, Kenya with the intent of improving maternal mental health and the socio-emotional development of young children. The program was based on content inclusive of cognitive behavioral therapy components and ECD messages. The primary objective of this study is to determine to what extent did the iMBC/ECD intervention implemented by community health volunteers affect maternal depression for mothers of young children. The secondary objective of the study is to determine to what extent did the iMBC/ECD intervention affect the social-emotional development of young children.

The findings of this study are expected to offer new insights on whether a community-based prevention model delivered by lay counselors in a low-resource setting can produce sustained effects on maternal mental health and children’s social and emotional development.

## 2. Methods

In 2018, Catholic Relief Services (CRS) implemented the Integrated Mothers and Babies Course and Early Childhood Development program (iMBC/ECD) (Le, 2018) within a larger donor-funded project, Strengthening the Capacity of Women Religious in Early Childhood Development (SCORE-ECD) in Siaya County, Kenya. The goal of SCORE-ECD was to provide high-quality community-based services to improve ECD outcomes by promoting health-seeking behaviors, nutrition for mothers and babies, water, sanitation and hygiene practices and early stimulation behaviors, as outlined in the Nurturing Care Framework (WHO et al., 2018).

The primary purpose of the curriculum-based Integrated Mothers and Babies Course (iMBC) is to reduce the symptoms and incidence of perinatal depression among vulnerable populations (Le et al., 2015). The theoretical underpinnings of this intervention are cognitive behavioral therapy and attachment theory with facilitated group sessions designed to equip participating women to manage common daily stressors that are relevant to the perinatal period, such as the mother-baby relationship, interpersonal and social relationships, common mood problems and unhealthy thought patterns, among others. The program offers practical skills on how to manage one’s thoughts, moods and relationships while encouraging active engagement in pleasant activities (Le, 2018; Muñoz et al., 2001). iMBC has been implemented among low-resource populations in the United States and has shown promising evidence of efficacy for lowering maternal depressive symptoms (Le et al., 2015). However, this program has not been evaluated in East Africa. The culturally adapted version of iMBC implemented in Kenya also incorporated key ECD messages regarding care of sick children, breastfeeding, nutrition for mothers and babies, water, sanitation and hygiene (WASH) practices, child protection, playing with children, early stimulation behaviors, and health services use, and thus was renamed iMBC/ECD. Although child development has various domains, the program implementors and evaluators anticipated that potential improvements in maternal mental health might most easily translate to improvements in socio-emotional development among their infants and young children, particularly given the focus on play and pleasant activities in the iMBC sessions.

The study built upon existing community-based *care groups* which were supported by CRS in the study areas. Care groups bring together women in communities via a network of community volunteers to deliver program content and are designed to be adopted by communities to produce sustainable change beyond donor funding cycles (Laughlin, 2010; Perry et al., 2015). Participants organized in care groups consisted primarily of pregnant women and mothers of children under two years old.

For the intervention areas, seven government community health volunteers (CHVs) delivered the iMBC/ECD content across a total of 23 care groups and monitored uptake of learned behaviors through home visits. The CHVs were supervised weekly by two Catholic sisters (nuns) and two CRS case managers who were trained as iMBC/ECD ‘master trainers’ and who monitored the CHVs performance using fidelity checklists. The CHVs were trained on iMBC/ECD prior to the intervention starting but master trainers also offered monthly mock sessions of iMBC/ECD *during* the intervention to reinforce the content and necessary facilitation skills. There were also “lead mothers” in the iMBC/ECD intervention groups but their role was largely focused on care group logistics (coordinating meeting dates, venue, home visit follow-ups for any women with issues).

For the control areas, there were 30 care groups and each group had one “lead mother” who was responsible for group logistics and delivering general ECD content aligning with nurturing care components (e. g. health, nutrition, responsive caregiving, security and safety, and early learning). Lead mothers conducted home visits to reinforce education messages and reported group and home visit attendance to their local CHV. CHVs conducted monthly supervision visits tailored to the challenges faced by lead mothers while supporting the group participants.

### 2.1. Study design, setting and participants

This quasi-experimental longitudinal study followed mother/baby dyads for nearly two years and utilized the existing care group model under SCORE-ECD for identifying the intervention and control groups. Assignment to intervention or control was by sub-county and not randomized due to issues of program logistics.

The intervention groups received iMBC/ECD content implemented over 14 in-person, group-based sessions spanning a period of 7 months (meetings occurring every two weeks). After completing the formal sessions, five iMBC/ECD follow-up booster sessions were conducted over the span of the next six months for a refresher on lessons learned during the program and to aid in practicing learned skills. The control groups received the same early childhood development education content during regular biweekly care group meetings as a part of the ongoing SCORE-ECD program. For both the intervention and control groups, care groups continued to meet beyond the duration of the study.

Women aged 16 years or older who planned to stay in the study area for the duration of the program and who were either pregnant or had a child under the age of two years old were recruited. At baseline, 417 women were enrolled and interviewed: 193 women in 23 intervention groups (Rarieda sub-county) and 224 women in 30 control groups (Ugunja sub-county).

Data were collected at four time points: baseline (August 2018), immediate post-intervention (July 2019; follow-up 1), 8-months post-intervention (January 2020; follow-up 2) and 14-months post-intervention (August 2020; follow-up 3). The year-long time lapse between baseline and the immediate post-intervention data collection was due to slight logistical delays in organizing data collection both before and after the iMBC/ECD program which ran from October 2018 to June 2019. Interviews lasted 60–90 min and were conducted in Luo by trained research assistants at locations conducive to confidential conversations in the community. Research assistants were also trained to provide referrals to participants who either endorsed suicidal ideation or self-harm and/or who reported experience of physical or sexual intimate partner violence in the past year. Participants had the option to accept or decline referrals. The last data collection period adhered to COVID-19 safety protocols per the IRB. The CommCare platform, published by Dimagi, Inc., was used to collect, store, and manage the study data.

To ensure implementation fidelity, program process data were collected and closely monitored throughout the duration of the program. For each of the 14 formal iMBC/ECD sessions, content coverage, time management, application of lessons learned, comprehension of the materials and level of participation were assessed. On a scale of 1 to 10, the master trainers rated observed selected sessions based on their perception of how well these five criteria were performed by the CHVs. Apart from the first three sessions, the remaining 11 sessions were consistently scored above 8 out of 10 for all five of the fidelity criteria used for this study (program data not shown).

### 2.2. Sample size and power calculation

Based on prior CRS programmatic data, we assumed that at pre-intervention, 15% of participants would meet the threshold for moderate/severe depression (PHQ-9 ≥ 10) in both groups, and at post-intervention, 5% of participants in the iMBC/ECD group would meet the threshold for moderate/severe depression (PHQ-9 ≥ 10) versus 15% in the ECD only control group. Using the SPA-ML program (Moerbeek and Teerenstra, 2016), we computed power under the following assumptions: two-sided alpha of 0.05, number of women per group of 6 (to allow for 25% dropout from 8 recruited per group), and number of groups per CHV of four. We assumed a very low ICC at the CHV level of 0.001, but a larger ICC of 0.01 at the group level. Thus, at 13 total CHVs (6 in one arm and 7 in the other), and 52 initially planned groups (24 in one arm and 28 in the other) we had a little over 70% power to detect this standardized effect size.

### 2.3. Primary outcome measures

Maternal depression was assessed using the Patient Health Questionnaire (PHQ-9) which has been previously validated in Kenya (Monahan et al., 2009). The PHQ-9 was forward and backward translated and pretested for the final Luo version. The PHQ-9 sum score ranging from 0 to 27 was analyzed as a continuous variable with higher scores indicated greater severity of depression. The Cronbach’s alpha was 0.83 at baseline, 0.84 at the immediate post-intervention follow-up, 0.83 at the 8-month post-intervention follow-up and 0.84 at the14-month post-intervention follow-up, indicating very good reliability.

Children’s social and emotional development was measured using the Ages and Stages Questionnaires: Social-Emotional, Second Edition (ASQ:SE-2) (Squires et al., 2015). The ASQ-SE consists of 16 to 36 caregiver-reported scored items, depending on child age, about the frequency of developmentally appropriate behaviors across the domains of self-regulation, compliance, social-communication, adaptive functioning, autonomy, affect, and interaction with people with responses ranging from ‘most of the time’ (0 points), ‘sometimes’ (5 points), and ‘rarely or never’ (10 points). Higher scores indicate greater concern about the child’s development. We used age-specific versions depending on the index child’s age (2-month, 6-month, 12-month, 18-month, 24-month, 30-month, 36-month, and 48-month) at data collection. They were forward translated into the Luo, reviewed by bilingual interviewers, and pretested prior to administration. Because different questionnaires (different number and type of questions) were administered depending on the child’s age, average item scores for each child at each time point were used to allow for comparison of all children across various age ranges. Average item scores were obtained by dividing the total sum score of the administered questionnaire by the total number of answered items on the same questionnaire. Higher average item scores indicated lower social and emotional development. Prior studies have used ASQ:SE-2 in low-resource settings (Krebs et al., 2011; Worku et al., 2018).

### 2.4. Covariates

Socio-demographic and health variables were examined as baseline characteristics and/or covariates in the analysis. Variables included maternal age, education, household size, parity, self-reported hope via the Herth Hope Index (Herth, 1991), the total score for mother-initiated early stimulation behaviors during pregnancy from factor analysis, maternal mental health score measured by the WHO’s Self-Reported Questionnaire (SRQ-20) (Beusenberg and Orley, 1994), the Household Hunger Scale (Ballard et al., 2011) and experience of physical, sexual or emotional violence or controlling behavior by husband/partner in the past year based on the DHS questionnaire (Croft et al., 2018). See Table 1 for a full list.

### 2.5. Statistical methods

**Fig. 1.**
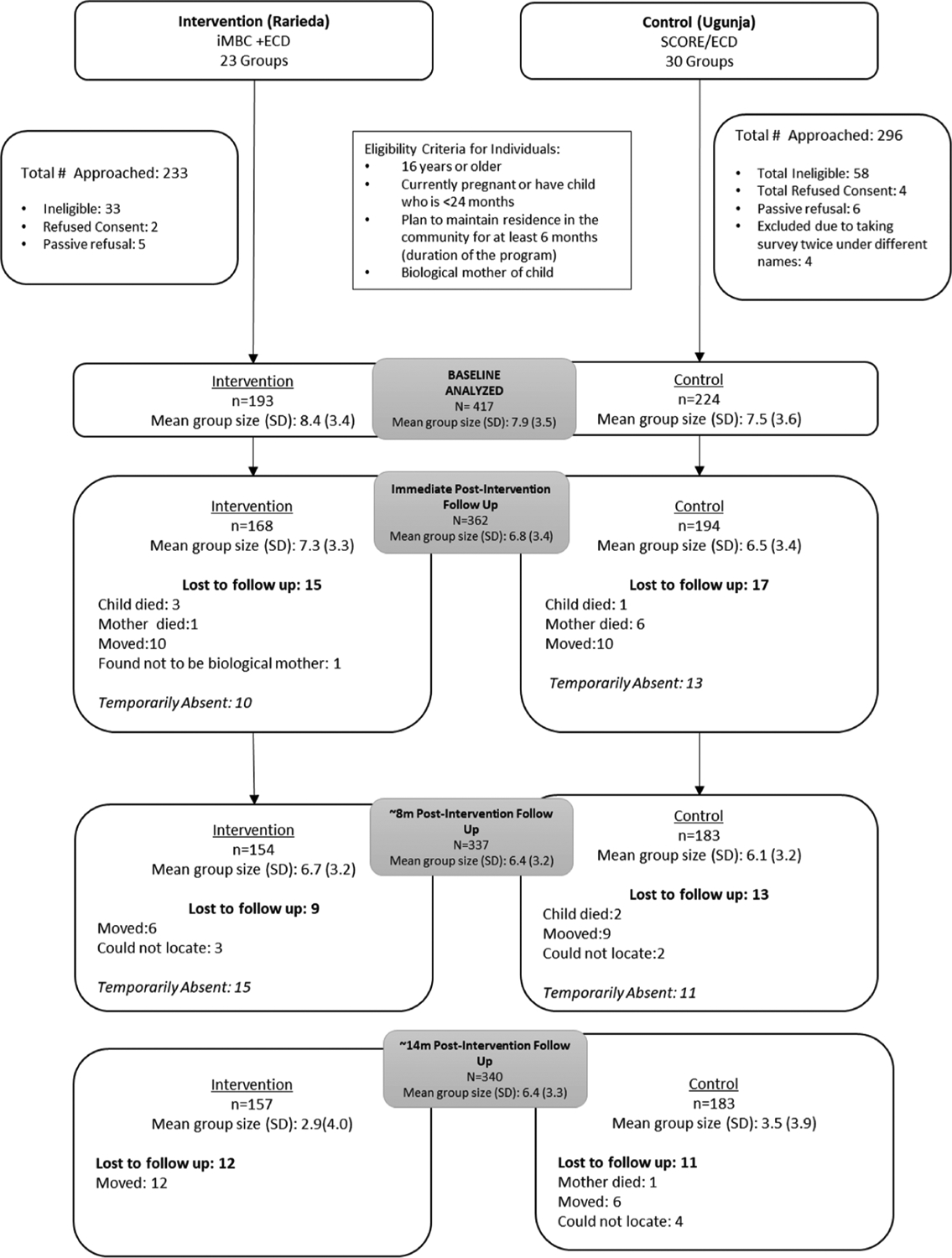
STROBE diagram.

STROBE guidelines were followed for analysis and presentation of results. A STROBE flow chart (von Elm et al., 2007) shows participation of intervention and control arm mothers and their children from recruitment through to 14 months post-intervention (Fig. 1). All analyses focused on comparison of the two arms. We also adhered to the latest guidance on presentation of p-values per the American Statistical Association (Wasserstein and Lazar, 2016).

Baseline maternal characteristics were reported by study arm. Primary analyses were intent-to-treat and conducted using Stata/SE 16.1 (StataCorp, College Station, Texas). The primary maternal outcome is PHQ-9 score at 14-month post-intervention follow-up (follow-up 3). The primary child outcome is the mean ASQ-SE item score also at follow-up 3. The mean item score was chosen to make the ASQ-SE comparable across different child age groups. To estimate the causal treatment effect from the non-randomized design, we implemented the propensity score (PS) weighting method to control for potential confounding factors. We estimated the PS using CBPS package in R (Imai and Ratkovic, 2014). Potential confounders included in the PS model were specified *a priori* (see Figures S1–S2 for a full list). Prior to PS estimation, single imputation of the missing values was performed and missingness indicators were included in the PS model to achieve balance in missingness patterns (Rosenbaum and Rubin, 1984). Both inverse probability weights (IPW) and overlap weights (OW) were considered (Hirano et al., 2003; Li et al., 2018), and IPW were used due to the observed level of overlap and balance in baseline covariates.

There was sufficient overlap of the distributions of estimated PSs in both the intervention and control arms, with the majority of the PSs distributed in the middle range (data not shown). The IPWs derived from the estimated PSs successfully reduced covariate imbalance (Figure S1). As for comparison of high attendance and low attendance group, which is defined below, the IPWs derived from the estimated PSs successfully reduced covariate imbalance as well (Figure S2).

Continuous primary outcomes were analyzed using linear mixed effects models so all comparisons of interest could be estimated from the same model. The identity link was used to estimate differences in mean outcomes. All mixed models included “care group” as a random intercept to account for the clustered study design. We also included a random personal-level intercept and random personal-level time slopes to account for the correlation of outcomes within person across time. We used maximum likelihood (ML) estimation, weighted by the pre-specified IPWs, with robust variance estimators to take weighting into account (Hernán and Robins, 2020, page 152). Direct regression models without PS weighting were fit for comparison.

Exploratory analyses examined if the effects of the intervention on the primary outcomes differed by the level of key covariates. PSs and balancing weights were calculated separately for each subgroup as recommended (Green and Stuart, 2014). As an additional exploratory analysis, we also re-fit the primary analysis models, comparing among participants in the intervention arm those attending more than half the sessions (high attendance) to those attending half or fewer sessions (low attendance; about 34.1% of participants). The control arm was not included in these analyses because control participants received the SCORE/ECD only programming, but no attendance data were available.

Lastly, to gain perspective on the implementation and acceptability of iMBC/ECD, 39 brief semi-structured interviews were conducted during the immediate post-intervention period in the intervention communities. Interviews included 20 iMBC/ECD participants, six lead mothers, and 13 people in leadership roles (CHVs, case managers, sister master trainers). Interviews were conducted in Luo or English, which-ever was preferred. A designated note taker accompanied the interviewers for extensive notetaking during the interview (no audio-recordings). Rapid content analysis was conducted using an analysis matrix in Microsoft Excel.

Ethical approval was received from the Duke University Campus IRB (# 2018–0595) and the Maseno University Ethics Review Committee in Kenya (# MUERC 00587/18). All participants provided written informed consent, or, if they were illiterate, were read the consent form and provided their fingerprint with a witness signature.

## 3. Results

### 3.1. Baseline characteristics

There were 417 participants in 53 groups at baseline (Table 1). Of these, 362 (86.8%), 337 (80.8%) and 340 (81.5%) participants were followed up at immediate post-intervention, 8-months post-intervention, and 14-months post-intervention, respectively. Reasons for loss-to-follow-up included maternal and infant mortality, maternal morbidity and migration, and marital separation, among others (Fig. 1). The average cluster size (care group) at baseline (standard deviation [SD]) was 7.5 (3.6) in control arm and 8.4 (3.4) in intervention arm. The average age of the women in the sample was 26.4 (5.5) years, with 60.4% of the sample having attended no more than primary school level and 40.7% having experienced physical/sexual abuse in the past year. At baseline, 10.3% of the total sample had indications of moderate to severe depression based on the PHQ-9 score. The mean PHQ-9 score in control arm [3.1 (3.6)] at baseline was slightly lower than that in the intervention arm [4.2 (4.6)] (Table 2).

### 3.2. Maternal mental health

There were no significant effects (confidence interval crosses the null) of the intervention on mean PHQ-9 scores. At immediate post-intervention follow-up, we observed a slightly larger but non-significant decrease in mean PHQ-9 score (i.e. improvement in maternal mental health) from baseline in the treatment arm than in the control arm [estimate (95% CI): −0.6 (–1.7, 0.5)]. At 8-month post-intervention, the difference in improvement from baseline was attenuated [estimate (95% CI): 0.1 (–1.2, 1.4)]. However, at 14-month post-intervention, we observed a larger but still non-significant decrease in mean PHQ-9 score from baseline in the intervention arm than in the control arm again [estimate (95% CI): −0.9 (–2.2, 0.4)]. See Table 3 and Figure S3.

### 3.3. Child socio-emotional development

There were no large effects of the intervention on mean ASQ-SE item scores. The average ASQ-SE mean item score at 8-month post-intervention was slightly higher in the intervention arm [estimate (95% CI): 0.2 (0.007, 0.5)]. However, the average ASQ-SE mean item score at 14-month post-intervention was slightly higher in the control arm [estimate (95% CI): −0.2 (–0.4, 0.1)]. See Table 3 and Figure S4.

Examining the individual ASQ-SE items descriptively, participants had the greatest concerns with issues of breastfeeding, soothing the child and diarrhea for infants under 12 months. Between 12 months and 48 months, participants’ greatest concerns were with children hurting themselves, hurting other children, adults, and animals, damaging things on purpose, excessive crying, repeating the same behavior and getting upset when trying to stop the child. Problems with eating was also a reported concern.

Unweighted regression results are similar to the estimated effects from the primary analyses (data not shown).

### 3.4. Exploratory analyses examining effects by participant attendance

In the analyses examining the effect of attendance in the intervention arm, in most cases this was not significant (confidence interval crossed the null) (See Table 4 and Figures S5–S6). The predicted mean PHQ-9 shows that a better improvement in maternal mental health persisted until 14-month follow-up period in the high-attendance group [estimate (95% CI): −1.8 (–2.9, 0.8)], while the improvement in low-attendance group decreased at the 14-month follow-up [estimate (95% CI): −0.2(–2.2, 1.9)]. Also, at the 14-month follow-up, the mean ASQ-SE item score was lower in the high-attendance group [estimate (95% CI): −0.4 (–0.9, −0.001)]. Twenty of the participants were excluded from this analysis due to missingness in the attendance variable.

### 3.5. Exploratory analyses examining effects modification

Effect modification analyses revealed that at the immediate post-intervention follow-up, the iMBC/ECD intervention was associated with: lower PHQ-9 scores (i.e. fewer symptoms of depression) for women with no education or only primary education [estimate (95% CI): −1.54 (–3.01, −0.07)]; lower PHQ-9 scores for women with four or more living children [estimate (95% CI): −2.75 (–5.49, 0.01)]; and higher ASQ-SE mean item scores (i.e. lower social and emotional development) overall and also for multiple sub-groups of women including younger-aged mothers and mothers who have experienced emotional intimate partner violence in the past year.

At the 14-month follow-up period, the iMBC/ECD intervention was associated with lower PHQ-9 scores for women who have not had any experience of physical or sexual intimate partner violence in the past year [estimate (95% CI):−1.93 (–3.75, −0.11)] or have not experienced controlling behavior by husbands/partners in the past year [estimate (95% CI): −2.28 (–4.46, −0.10)]. The iMBC/ECD intervention was also associated with higher ASQ-SE mean item scores overall. See Figures S7–S12 for other significant effect modifiers.

### 3.6. Key qualitative findings

Almost all iMBC/ECD participants mentioned having had a positive experience, particularly regarding the opportunity to share with other mothers about their life circumstances and using the contextualized storytelling method. Some participants mentioned that communication with their husbands/partners had improved and the majority of the participants also indicated that they were strongly supportive of husbands/partners involvement in some version of the iMBC/ECD program, including discussions about couple’s relationship dynamics and ECD. With regards to iMBC program content specifically, some leaders indicated that participants expressed difficulty implementing some cognitive-behavior therapy practices such as identifying and defining thought patterns and practicing “thought interruption”, where one replaces a negative thought pattern with a positive thought. Participants seemed to have more traction with understanding and practicing lessons related to pleasant activities and planning day-to-day activities. Participants also expressed that the iMBC/ECD program could benefit from including economic strengthening activities.

## 4. Discussion

Findings of this evaluation provide valuable programmatic insights for future community-based, integrated caregiver mental health and ECD interventions in low-resource settings. Overall, the iMBC/ECD intervention did not have a significant impact on decreasing maternal depression symptoms or on increasing the socio-emotional development of children compared to the ECD only control group. It is important to note however that the study did not specifically target women with depression. Rather, this was a community-based, prevention-oriented intervention, hence the sample had relatively low mean PHQ-9 scores at all time points. This was true even with the occurrence of the COVID-19 pandemic before the last data collection time point in August 2020. Moreover, evaluations of prevention-oriented mental health interventions are still nascent in low-resource settings and further studies are needed to understand their impact and potential (Wainberg et al.,2017).

The lack of measured effect begs the question of whether iMBC was appropriate for the context. The largely picture-based iMBC program was steeped in cognitive behavioral therapy (CBT) and the literature regarding the efficacy of CBT for a range of specific mental and behavioral problems is strong, although largely based on studies in high-income countries (Hofmann et al., 2012; Cuijpers et al., 2018). That said, CBT is not the only theoretical model for treating depression. For example, a recent review of psychological treatments among people living with HIV/AIDS in LMICs revealed that CBT showed more modest effectiveness in reducing depressive symptoms compared to other models such as problem-solving therapy and interpersonal therapy (Asrat et al., 2020). The review hypothesized that since CBT typically requires more advanced skills training and more sessions than other therapies, perhaps this contributed to their lower effect estimates. While our intervention was not aimed at HIV-affected populations, the review does point out that not all psychological treatments for depression that are evidence-based in high-income country settings, will have necessarily have similar effectiveness in LMIC settings. Sub-group analyses in our study indicated that the iMBC/ECD program, relative to the control group, was associated with lowered depressive symptoms among specific sub-groups of women such as those with low levels of education and those with four or more children. A cluster-randomized controlled trial of iMBC/ECD implemented in Ghana also did not see overall significant effects but did see impacts on certain more vulnerable subgroups (Baumgartner et al., 2021).

Despite the program including participant role play and using real-life scenarios tailored for Kenya via storytelling, participants’ difficulty with components of CBT exercises such as ‘though interruption’ may have affected overall program effectiveness. The program team performed internal checks (i.e. sister master trainers using fidelity forms) to ensure that the facilitators implemented each program session as intended; however, it is still plausible that there was a disconnect between lay community health volunteers (CHVs) delivering iMBC content with ‘fidelity’ and participants receiving in the information in a way that changed their feelings, thoughts, and/or behaviors. Likewise, general challenges with task-shifting have been reported in other settings (Mundeva et al., 2018; Okyere et al., 2017) and there are still many unknown barriers and facilitators to optimal delivery of task-shifting interventions for perinatal depression in low- and middle-income countries (Munodawafa et al., 2018).

Another reason for the lack of an overall program impact may have been because all study participants were part of care groups and these groups, regardless of content, had an unexpected positive effect on mental health. It is plausible that just being a part of a regular support group with other women in similar life stages and having a sense of belonging may positively influence their mental health. However, this is largely speculation and we call for future studies to investigate this topic further.

Program attendance mattered for program impact. Women with high program attendance (attended more than half of sessions) experienced consistently fewer depression symptoms compared to those with low program attendance (attending half or fewer sessions). This finding is consistent with a recent review outlining programmatic guidance for ECD interventions in high HIV burden countries (Tomlinson et al., 2020). The review found that successful programs reported high levels of attendance and a total duration of at least six months [iMBC/ECD was 7 month long] (Tomlinson et al., 2020). It is important to note that despite having achieved sufficient covariate balance on important variables, we exercise caution not to fully attribute lower depression symptoms to high program attendance because of the possibility that omitted factors potentially driving program attendance also correlate with participants’ mental health.

Regarding the intervention’s lack of desired impact on children’s socio-emotional development (i.e. ASQ-SE mean item scores were slightly higher in the intervention group compared to the control group), the findings must be interpreted in the context that the mean ASQ-SE item scores were generally low at all follow-up time points in the study (indicating general good development) and the difference in the ASQ-SE mean item scores between the intervention and control groups at the follow-up time points were miniscule. This perhaps signified that there was little room for developmental improvement in the children of our study sample by the intervention. It is also important to note that ASQ-SE was initially designed for screening purposes and not necessarily for longitudinal research. Regardless, we generally recommend that an integrated ECD curriculum should include multiple didactic and interactive practice sessions that focus on age-appropriate responsive caregiving and feeding, early stimulation and positive discipline.

In order for future programs to potentially have broader community-wide impact, the existing iMBC/ECD curriculum may need to be integrated with components that more directly address the socioeconomic determinants of mental health at the individual, familial and/or community levels (World Health Organization, 2014). For example, economic strengthening activities, as noted by iMBC/ECD participants during qualitative interviews, that help alleviate household poverty and reduce hunger could be critical as such activities show evidence to be protective of caregiver mental health (Wang et al., 2014).

In addition, intimate partner violence cannot be overlooked in community-based mental health and early child development programming. We found that iMBC/ECD, relative to the control group, was associated with lowered depressive symptoms among women who have not had any experience of physical or sexual intimate partner violence or marital control in the past year. This suggests that without proactively addressing intimate partner violence, cognitive behavior therapy alone may not be enough when persistent mental health problems are primarily driven by difficult relational dynamics and abuses that are beyond the women’s control. As such, involving male partners, with the women’s consent, in community-based intervention models could be a key strategy moving forward. Past studies have highlighted the value of male involvement in improving maternal mental health and early child development interventions (Jeong et al., 2016; Tokhi et al., 2018; Yargawa and Leonardi-Bee, 2015). Another study specifically highlighted the value of male involvement in community-based intimate partner violence prevention efforts and called for more of these in future programming (Starmann et al., 2017). In addition, many of the iMBC/ECD participants were favorable to their partners being involved in the program as well. Active male involvement and endorsement for the program could potentially increase program attendance.

There have not been many interventions designed to address both caregiver mental health and ECD in an integrated manner in low-resource settings (Maselko et al., 2015; Singla et al., 2015; Turner et al., 2016). The current iMBC/ECD program is unique in that it is a low-cost, community-facilitated, integrated caregiver mental health and ECD intervention that is designed to be implemented by non-specialists. There are many benefits to utilizing a community-based intervention design: greater acceptability by the recipients, greater accessibility to services compared to facility-based services, increased capacity to monitor ongoing progress, existing rapport with trusted community-based providers and the potential for greater family involvement (Kohrt et al., 2018). More recently, there has also been efforts to use a peer-to-peer delivery mechanism (Atif et al., 2017; Sikander et al., 2015; Turner et al., 2016; Vanobberghen et al., 2020). Although we did not see dramatic reductions in maternal depression and substantial improvements in children’s socio-emotional development with iMBC/ECD implemented at the community level, there were some promising signs for certain vulnerable sub-groups and when high program attendance could be achieved. With consideration for these sub-groups in tandem with high attendance, iMBC/ECD holds promise in this setting.

There are study limitations. First, this was not a cluster-randomized controlled trial. Rather, two sub-counties were purposefully selected and the iMBC/ECD program was rolled out in one of the counties. Despite this limitation, application of the IPWs using covariate balancing PSs created a weighted target population in which women in the intervention and control groups were comparable, at least on a set of observed covariates included in the estimation of the PSs. Second, even though balance was sufficiently achieved on a set of important observed covariates, the control group may not have been a true control to the intervention group. Third, this study was likely underpowered to assess the program’s effect on maternal mental health and children’s social and emotional development because initial expectations were that the level of moderate/severe depression (PHQ-9 ≥ 10) would be higher than they actually were. That said, there were no noticeable practical differences in depression scores that would have influenced our conclusions. Findings related to sub-group analyses (effect modification) and supplemental analyses comparing women with high versus low attendance are also exploratory and not powered. Nonetheless, we believe that these additional analyses provide helpful programmatic insights and nuanced understandings of the main findings.

## 5. Conclusion

Overall, the iMBC/ECD program did not have a measurable impact on our primary outcomes at a population level; however, we note that this study was in a context where the majority of participants did not show indications of moderate to severe depression and their children had favorable developmental scores from the onset. With this in mind, the program may have the potential to improve maternal mental health and early child development for more targeted vulnerable populations (e.g. those with depression at program enrollment, lower education, higher parity) and under specific conditions (e.g. implementation fidelity is assured, attendance is high, and ample time is devoted to specific ECD content). Going forward, programs with the dual purpose of improving maternal mental health and child development and their associated evaluations should consider the implications of a non-targeted, community-level approach versus a more targeted approach that may optimize impact. Likewise, these integrated programs should also consider including components that address physical or sexual intimate partner violence or marital control and the socioeconomic determinants of mental health. Further research is still needed to identify effective, integrated programming in support of responsive caregiving in order to ensure that all mothers and their children thrive.

## Supplementary Material

1

## Figures and Tables

**Table 1:** Baseline characteristics of study population, Siaya County, Kenya.

	Control (*N* = 224)	Intervention (*N* = 193)	Total (*N* = 417)
**Age in Years**			
Mean (SD)	26.59 (5.51)	26.14 (5.57)	26.38 (5.54)
Min, Max	17.0, 42.0	17.0, 42.0	17.0, 42.0
**Number of Times Pregnant**			
Mean (SD)	3.29 (1.71)	3.15 (1.79)	3.22 (1.74)
Min, Max	1.0, 9.0	1.0, 10.0	1.0, 10.0
**PHQ-9 Score**			
Mean (SD)	3.09 (3.64)	4.23 (4.58)	3.62 (4.14)
Min, Max	0.0, 16.0	0.0, 21.0	0.0, 21.0
**PHQ-9 Categorized**			
None/Minimal (0–4)	162 (72.3%)	124 (64.2%)	286 (68.6%)
Mild (5–9)	45 (20.1%)	43 (22.3%)	88 (21.1%)
Moderate (10–14)	13 (5.8%)	18 (9.3%)	31 (7.4%)
Moderately Severe (15–19)	4 (1.8%)	7 (3.6%)	11 (2.6%)
Severe (20–27)	0 (0.0%)	1 (0.5%)	1 (0.2%)
**PHQ-9 Dichotomized**			
None to Mild (0–9)	207 (92.4%)	167 (86.5%)	374 (89.7%)
Moderate to Severe (>10)	17 (7.6%)	26 (13.5%)	43 (10.3%)
**SRQ-20 Score**			
Mean (SD)	4.05 (4.15)	5.32 (4.63)	4.64 (4.42)
Min, Max	0.0, 18.0	0.0, 19.0	0.0, 19.0
**Herth Hope Index Score**			
Mean (SD)	35.57 (2.99)	35.74 (3.56)	35.65 (3.26)
Min, Max	26.0, 47.0	19.0, 48.0	19.0, 48.0
**Household Size (# children + adults)**			
Mean (SD)	5.37 (1.74)	5.63 (2.08)	5.49 (1.91)
Min, Max	2.0, 11.0	1.0, 14.0	1.0, 14.0
**Hunger Score: Categorized**			
little to none	185 (82.6%)	159 (82.4%)	344 (82.5%)
moderate	34 (15.2%)	28 (14.5%)	62 (14.9%)
Severe hunger	5 (2.2%)	6 (3.1%)	11 (2.6%)
**Highest Level of School Attended**			
No education	3 (1.3%)	2 (1.0%)	5 (1.2%)
Primary	132 (58.9%)	115 (59.6%)	247 (59.2%)
Post-primary	16 (7.1%)	10 (5.2%)	26 (6.2%)
Secondary/A level	60 (26.8%)	53 (27.5%)	113 (27.1%)
College (midlevel)	9 (4.0%)	12 (6.2%)	21 (5.0%)
University	2 (0.9%)	1 (0.5%)	3 (0.7%)
Data missing	2 (0.9%)	0 (0.0%)	2 (0.5%)
**Women’s Self-Reported Health**			
Excellent	15 (6.7%)	13 (6.7%)	28 (6.7%)
Very Good	38 (17.0%)	36 (18.7%)	74 (17.7%)
Good	121 (54.0%)	105 (54.4%)	226 (54.2%)
Fair	44 (19.6%)	35 (18.1%)	79 (18.9%)
Poor	6 (2.7%)	4 (2.1%)	10 (2.4%)
**Child’s Health Reported by Mother**			
Fair/Poor	31 (15.2%)	19 (11.2%)	50 (13.4%)
Good	120 (58.8%)	105 (61.8%)	225 (60.2%)
Very Good/Excellent	53 (26.0%)	46 (27.1%)	99 (26.5%)
Women still pregnant	20	23	43
**Low Birth Weight (<2500 g)**			
Normal Weight	194 (95.1%)	160 (94.1%)	354 (94.7%)
Low Birth Weight	10 (4.9%)	7 (4.1%)	17 (4.5%)
Data missing	0 (0.0%)	3 (1.8%)	3 (0.8%)
Women still pregnant	20	23	43
**Currently breastfeeding child**			
No	27 (13.2%)	22 (12.9%)	49 (13.1%)
Yes	175 (85.8%)	145 (85.3%)	320 (85.6%)
Data missing	2 (1.0%)	3 (1.8%)	5 (1.3%)
Women still pregnant	20	23	43
**Social Support-Husband (in the past month)**			
No	88 (41.1%)	66 (36.3%)	154 (38.9%)
Yes	121 (56.5%)	105 (57.7%)	226 (57.1%)
Data missing	5 (2.3%)	11 (6.0%)	16 (4.0%)
No partner	10	11	21
**Social Support-Female relatives (in the past month)**			
Never/Insufficient	159 (71.0%)	130 (67.4%)	289 (69.3%)
Sufficient	65 (29.0%)	62 (32.1%)	127 (30.5%)
Data missing	0 (0.0%)	1 (0.5%)	1 (0.2%)
**Social Support-Female friends (in the past month)**			
Never/Insufficient	171 (76.3%)	138 (71.5%)	309 (74.1%)
Sufficient	53 (23.7%)	54 (28.0%)	107 (25.7%)
Data missing	0 (0.0%)	1 (0.5%)	1 (0.2%)
**Relationship Status**			
Married and living with husband	186 (83.0%)	142 (73.6%)	328 (78.7%)
Married and not living with husband	14 (6.3%)	11 (5.7%)	25 (6.0%)
Living with romantic partner whom you are not married to	1 (0.4%)	0 (0.0%)	1 (0.2%)
With romantic partner but not married nor living together	9 (4.0%)	24 (12.4%)	33 (7.9%)
Not Currently in a relationship	14 (6.3%)	16 (8.3%)	30 (7.2%)
**Have you done any work in the last 7 days**			
No	139 (62.1%)	124 (64.2%)	263 (63.1%)
Yes	85 (37.9%)	69 (35.8%)	154 (36.9%)
**Anyone in household has HIV/AIDS**			
No	169 (75.4%)	143 (74.1%)	312 (74.8%)
Yes	55 (24.6%)	49 (25.4%)	104 (24.9%)
Data missing	0 (0.0%)	1 (0.5%)	1 (0.2%)
**Physical/Sexual Violence by Husband/Partner in the Past Year**			
No	100 (46.7%)	72 (39.6%)	172 (43.4%)
Yes	86 (40.2%)	75 (41.2%)	161 (40.7%)
Data missing	28 (13.1%)	35 (19.2%)	63 (15.9%)
No partner	10	11	21
**Emotional Violence by Husband/Partner in the Past Year**			
No	142 (66.4%)	105 (57.7%)	247 (62.4%)
Yes	46 (21.5%)	41 (22.5%)	87 (22.0%)
Data missing	26 (12.1%)	36 (19.8%)	62 (15.7%)
No partner	10	11	21
**Controlling Behavior by Husband/Partner in the Past Year**			
No	93 (43.5%)	57 (31.3%)	150 (37.9%)
Yes	92 (43.0%)	86 (47.3%)	178 (44.9%)
Data missing	29 (13.6%)	39 (21.4%)	68 (17.2%)
No partner	10	11	21
**Factor Score for Early Stimulation Behaviors (ESB) during Pregnancy**			
Mean (SD)	5.54 (2.50)	4.56 (1.88)	5.09 (2.28)
Min, Max	3.1, 12.4	3.1, 12.4	3.1, 12.4
**During current pregnancy do you: Talk softly to him/her & touch belly?**			
Never	96 (42.9%)	116 (60.1%)	212 (50.8%)
Rarely	37 (16.5%)	30 (15.5%)	67 (16.1%)
Sometimes	37 (16.5%)	23 (11.9%)	60 (14.4%)
Frequently	52 (23.2%)	23 (11.9%)	75 (18.0%)
Data missing	2 (0.9%)	1 (0.5%)	3 (0.7%)
**During current pregnancy do you: Sing songs to him/her?**			
Never	136 (60.7%)	147 (76.2%)	283 (67.9%)
Rarely	26 (11.6%)	24 (12.4%)	50 (12.0%)
Sometimes	41 (18.3%)	13 (6.7%)	54 (12.9%)
Frequently	20 (8.9%)	8 (4.1%)	28 (6.7%)
Data missing	1 (0.4%)	1 (0.5%)	2 (0.5%)
**During current pregnancy do you: Tell him/her about his/her family?**			
Never	160 (71.4%)	163 (84.5%)	323 (77.5%)
Rarely	31 (13.8%)	20 (10.4%)	51 (12.2%)
Sometimes	21 (9.4%)	7 (3.6%)	28 (6.7%)
Frequently	11 (4.9%)	2 (1.0%)	13 (3.1%)
Data missing	1 (0.4%)	1 (0.5%)	2 (0.5%)
**During current pregnancy do you: Dance to music or the radio?**			
Never	127 (56.7%)	125 (64.8%)	252 (60.4%)
Rarely	41 (18.3%)	29 (15.0%)	70 (16.8%)
Sometimes	31 (13.8%)	23 (11.9%)	54 (12.9%)
Frequently	24 (10.7%)	15 (7.8%)	39 (9.4%)
Data missing	1 (0.4%)	1 (0.5%)	2 (0.5%)
**During current pregnancy do you: Encourage older children to touch &talk?**			
Never	159 (71.0%)	155 (80.3%)	314 (75.3%)
Rarely	26 (11.6%)	23 (11.9%)	49 (11.8%)
Sometimes	26 (11.6%)	10 (5.2%)	36 (8.6%)
Frequently	11 (4.9%)	2 (1.0%)	13 (3.1%)
Data missing	2 (0.9%)	3 (1.6%)	5 (1.2%)
**During current pregnancy do you: Encourage partner/husband to talk & touch?**			
Never	105 (46.9%)	130 (67.4%)	235 (56.4%)
Rarely	45 (20.1%)	27 (14.0%)	72 (17.3%)
Sometimes	39 (17.4%)	19 (9.8%)	58 (13.9%)
Frequently	34 (15.2%)	16 (8.3%)	50 (12.0%)
Data missing	1 (0.4%)	1 (0.5%)	2 (0.5%)

**Table 2:** Primary outcomes.

	Control (*N* = 224)	Intervention (*N* = 193)	p-value
**ASQ-SE Score, Follow-up 1**			0.009
Mean (SD)	1.74 (0.87)	2.04 (1.05)	
N (% Non-missing)	179 (79.9%)	154 (79.8%)	
**ASQ-SE Score, Follow-up 2**			0.025
Mean (SD)	1.71 (0.94)	1.97 (0.92)	
N (% Non-missing)	183 (81.7%)	154 (79.8%)	
**ASQ-SE Score, Follow-up 3**			0.552
Mean (SD)	2.00 (1.23)	1.92 (1.01)	
N (% Non-missing)	183 (81.7%)	157 (81.3%)	
**PHQ-9 Score, Baseline**			0.024
Mean (SD)	3.09 (3.64)	4.23 (4.58)	
N (% Non-missing)	224 (100.0%)	193 (100.0%)	
**PHQ-9 Score, Follow-up 1**			0.645
Mean (SD)	2.63 (3.99)	2.82 (3.74)	
N (% Non-missing)	194 (86.6%)	168 (87.0%)	
**PHQ-9 Score, Follow-up 2**			0.071
Mean (SD)	2.42 (3.49)	3.21 (4.07)	
N (% Non-missing)	183 (81.7%)	154 (79.8%)	
**PHQ-9 Score, Follow-up 3**			0.899
Mean (SD)	2.86 (3.84)	2.85 (3.55)	
N (% Non-missing)	183 (81.7%)	157 (81.3%)	
**Change in PHQ-9 from baseline to follow-up 1**			0.045
Mean (SD)	−0.27 (4.40)	−1.43 (5.46)	
N (% Non-missing)	194 (86.6%)	168 (87.0%)	
**Change in PHQ-9 from baseline to follow-up 2**			0.645
Mean (SD)	−0.71 (4.29)	−0.98 (5.48)	
N (% Non-missing)	183 (81.7%)	154 (79.8%)	
**Change in PHQ-9 from baseline to follow-up 3**			0.044
Mean (SD)	−0.15 (4.63)	−1.43 (5.42)	
N (% Non-missing)	183 (81.7%)	157 (81.3%)	
**PHQ-9 Dichotomized, Baseline**			0.133
None to Mild (0–9)	207 (92.4%)	167 (86.5%)	
Moderate to Severe (>10)	17 (7.6%)	26 (13.5%)	
**PHQ-9 Dichotomized, Follow-up 1**			0.831
None to Mild (0–9)	179 (92.3%)	156 (92.9%)	
Moderate to Severe (>10)	15 (7.7%)	12 (7.1%)	
**PHQ-9 Dichotomized, Follow-up 2**			0.043
None to Mild (0–9)	176 (96.2%)	139 (90.3%)	
Moderate to Severe (>10)	7 (3.8%)	15 (9.7%)	
**PHQ-9 Dichotomized, Follow-up 3**			0.785
None to Mild (0–9)	169 (92.3%)	146 (93.0%)	
Moderate to Severe (>10)	14 (7.7%)	11 (7.0%)	

Note. The dichotomized PHQ-9 were presented as supporting information for programmatic purposes. Only the continuous PHQ-9 scores were used in regression analyses.

**Table 3:** Continuous regression results for mean PHQ-9 score and mean ASQ-SE score.

Maternal mental health	Predicted mean PHQ-9 in treatment group	Predicted mean PHQ-9 in usual care group	Predicted mean change in PHQ-9 from baseline, treatment group	Predicted mean change in PHQ-9 from baseline, control group	Predicted mean difference in change from baseline, treatment vs. control
Baseline	3.9 (3.2, 4.6)	3.3 (2.6, 4.0)			
Follow-up 1	2.8 (2.3, 3.3)	2.8 (2.2, 3.4)	−1.1 (−1.9, −0.4)	−0.6 (−1.4, 0.2)	**−0.6 (−1.7, 0.5)**
Follow-up 2	3.3 (2.4, 4.2)	2.6 (2.1, 3.1)	−0.7 (−1.8, 0.4)	−0.8 (−1.5, −0.1)	**0.1 (−1.2, 1.4)**
Follow-up 3	2.7 (1.9, 3.5)	3.0 (2.4, 3.6)	−1.3 (−2.2, −0.3)	−0.3 (−1.2, 0.6)	**−0.9 (−2.2, 0.4)**
**Child Socio-emotional development**	Predicted mean ASQ-SE in treatment group	Predicted mean ASQ-SE in usual care group			**Predicted mean difference, treatment vs. control**
Follow-up 1	2.0 (1.8, 2.2)	1.7 (1.5, 1.9)			**0.3 (0.05, 0.5)**
Follow-up 2	1.9 (1.7, 2.1)	1.7 (1.5, 1.9)			**0.2 (0.007, 0.5)**
Follow-up 3	1.9 (1.7, 2.1)	2.0 (1.8, 2.2)			**−0.2 (−0.4, 0.1)**

**Table 4:** Exploratory analysis (high attendance vs low attendance): continuous regression results for mean PHQ-9 score and mean ASQ-SE score.

Maternal mental health	Predicted mean PHQ-9 in high attendance group	Predicted mean PHQ-9 in low attendance group	Predicted mean change in PHQ-9 from baseline, high attendance group	Predicted mean change in PHQ-9 from baseline, low attendance group	Predicted mean difference in change from baseline, high vs. low
Baseline	4.2 (3.2, 5.2)	3.8 (2.6, 5.0)			
Follow-up 1	2.4 (1.8, 3.0)	3.3 (2.1, 4.5)	−1.8 (−2.8, −0.8)	−0.5 (−1.6, 0.7)	**−1.3 (−3.0, 0.3)**
Follow-up 2	2.5 (1.5, 3.5)	3.8 (2.3, 5.3)	−1.7 (−2.8, −0.5)	0.02 (−1.7, 1.7)	**−1.7 (−3.7, 0.3)**
Follow-up 3	2.4 (1.5, 3.3)	3.6 (2.1, 5.1)	−1.8 (−2.9, −0.8)	−0.2 (−2.2, 1.9)	**−1.7 (−3.9, 0.6)**
**Child Socio-emotional development**	Predicted mean ASQ-SE in high attendance group	Predicted mean ASQ-SE in low attendance group			**Predicted mean difference, high vs. low**
Follow-up 1	2.0 (1.8, 2.2)	1.9 (1.6, 2.2)			**0.1 (−0.3, 0.4)**
Follow-up 2	1.9 (1.7, 2.1)	2.0 (1.7, 2.3)			**−0.1 (−0.5, 0.3)**
Follow-up 3	1.8 (1.6, 2.0)	2.2 (1.9, 2.5)			**−0.4 (−0.9, −0.001)**

## References

[R1] Asrat B, Schneider M, Ambaw F, Lund C, 2020. Effectiveness of psychological treatments for depressive symptoms among people living with HIV/AIDS in low- and middle-income countries: a systematic review and meta-analysis. J. Affect. Disord 270, 174–187. 10.1016/j.jad.2020.03.068. 32339109

[R2] Atif N, Krishna RN, Sikander S, Lazarus A, Nisar A, Ahmad I, Raman R, Fuhr DC, Patel V, Rahman A, 2017. Mother-to-mother therapy in India and Pakistan: adaptation and feasibility evaluation of the peer-delivered thinking healthy programme. BMC Psychiatry 17 (1), 79. 10.1186/s12888-017-1244-z. 28231791 PMC5324237

[R3] Ballard T, Coates J, Swindale A, & Deitchler M (2011). Household Hunger Scale: indicator Definition and Measurement Guide.

[R4] Baumgartner JN, Ali M, Gallis J, Lillie M, Abubakr-Bibilazu S, Adam H, Aborigo R, Owusu R, Kim E, McEwan E, Zhou Y, Mackness J, Awoonor-Williams J, Hembling J, 2021. Effect of a lay counselor delivered integrated maternal mental health and early childhood development group-based intervention in Northern Ghana: a cluster randomized controlled trial. Global Mental Health 8 (E18). 10.1017/gmh.2021.15 [in press]. PMC8157813 34104458

[R5] Beusenberg M, & Orley J (1994). A User’s Guide to the Self Reporting Questionnaire (SRQ).

[R6] Black MM, Walker SP, Fernald LCH, Andersen CT, DiGirolamo AM, Lu C, McCoy DC, Fink G, Shawar YR, Shiffman J, Devercelli AE, Wodon QT, Vargas-Barón E, Grantham-McGregor S, 2017. Early childhood development coming of age: science through the life course. Lancet 389, 77–90. 10.1016/S0140-6736(16)31389-7. 27717614 PMC5884058

[R7] Chan M, Lake A, Hansen K, 2016. The early years: silent emergency or unique opportunity? Lancet 11–13. 10.1016/S0140-6736(16)31701-9. 27717612

[R8] Croft T, Marshall A, & Allen C (2018). Guide to DHS Statistics.

[R9] Cuijpers P, Karyotaki E, Reijnders M, Purgato M, Barbui C, 2018. Psychotherapies for depression in low- and middle-income countries: a meta-analysis. World Psychiatry 17 (1), 90–101. 10.1002/wps.20493. 29352530 PMC5775122

[R10] Daelmans B, Darmstadt GL, Lombardi J, Black MM, Britto PR, Lye S, Dua T, Bhutta ZA, Richter LM, 2016. Early childhood development: the foundation of sustainable development. Lancet 9–11. 10.1016/S0140-6736(16)31659-2. 27717607

[R11] Fisher J, de Mello MC, Patel V, Rahman A, Tran T, Holton S, Holmes W, 2012. Prevalence and determinants of common perinatal mental disorders in women in low-and lower-middle-income countries: a systematic review. Bull. World Health Organ 90, 139–149. 10.2471/BLT.11.091850. PMC3302553 22423165

[R12] Gray K, Frey M, & Schwethelm B (n.d. 2021). Scaling up nurturing care in Siaya County, Kenya.

[R13] Green KM, Stuart EA, 2014. Examining moderation analyses in propensity score methods: application to depression and substance use. J. Consult. Clin. Psychol 82 (5), 773–783. 10.1037/a0036515. 24731233 PMC4172552

[R14] Herba CM, Glover V, Ramchandani PG, Rondon M, 2016. Maternal depression and mental health in early childhood: an examination of underlying mechanisms in low-income and middle-income countries. Lancet Psychiatry 3 (10), 983–992. 10.1016/S2215-0366(16)30148-1. 27650772

[R15] Hernán M, Robins J, 2020. Causal Inference: What If. Chapman & Hall/CRC, Boca Raton.

[R16] Herth K, 1991. Development and refinement of an instrument to measure hope. Sch. Inq. Nurs. Pract 5 (1), 39–51. 2063043

[R17] Hirano K, Imbens GW, Ridder G, 2003. Efficient estimation of average treatment effects using the estimated propensity score. Econometrica 71 (4), 1161–1189.

[R18] Hofmann SG, Asnaani A, Vonk IJJ, Sawyer AT, Fang A, 2012. The efficacy of cognitive behavioral therapy: a review of meta-analyses. Cognit. Ther. Res 36 (5), 427–440. 10.1007/s10608-012-9476-1. PMC3584580 23459093

[R19] Imai K, Ratkovic M, 2014. Covariate balancing propensity score. J. R. Stat. Soc 76 (1), 243–263. 10.1111/rssb.12027.

[R20] Jeong J, McCoy DC, Yousafzai AK, Salhi C, Fink G, 2016. Paternal stimulation and early child development in low-and middle-income countries. Pediatrics 138 (4), e20161357. 10.1542/peds.2016-1357. 27600319

[R21] Kenya Ministry of Health. (2020). Kenya Community Health Policy 2020–2030.

[R22] Kohrt BA, Asher L, Bhardwaj A, Fazel M, Jordans MJD, Mutamba BB, Nadkarni A, Pedersen GA, Singla DR, Patel V, 2018. The role of communities in mental health care in low-and middle-income countries: a meta-review of components and competencies. Int. J. Environ. Res. Public Health 15 (6), 1279. 10.3390/ijerph15061279. PMC6025474 29914185

[R23] Krebs NF, Hambidge KM, Mazariegos M, Westcott J, Goco N, Wright LL, Koso-Thomas M, Tshefu A, Bose C, Pasha O, Goldenberg R, Chomba E, Carlo W, Kindem M, Das A, Hartwell T, McClure E, 2011. Complementary feeding: a Global Network cluster randomized controlled trial. BMC Pediatr. 11 (4) 10.1186/1471-2431-11-4. PMC3032692 21232139

[R24] Laughlin M, 2010. The Care Group difference: a guide to mobilizing community-based volunteer health educators. 2nd ed. Baltimore (MD): World Relief. Co-published by CORE Group. Available from: https://coregroup.org/wp-content/uploads/media-backup/documents/Resources/Tools/Care_Group_Manual_Final__Oct_2010.pdf.

[R25] Le H-N (2018). The Integrated Mothers and Babies Course Facilitator’s Manual.

[R26] Le H-N, Perry DF, Mendelson T, Tandon SD, Muñoz RF, 2015. Preventing perinatal depression in high risk women: moving the mothers and babies course from clinical trials to community implementation. Matern. Child Health J 19 (10), 2102–2110. 10.1007/s10995-015-1729-7. 25673369

[R27] Li F, Morgan KL, Zaslavsky AM, 2018. Balancing covariates via propensity score weighting. J Am Stat Assoc 113 (521), 390–400. 10.1080/01621459.2016.1260466.

[R28] Machel G, 2016. Good early development—the right of every child. Lancet 13–14. 10.1016/S0140-6736(16)31700-7. 27717608

[R29] Maselko J, Sikander S, Bhalotra S, Bangash O, Ganga N, Mukherjee S, Egger H, Franz L, Bibi A, Liaqat R, Kanwal M, Abbasi T, Noor M, Ameen N, Rahman A, 2015. Effect of an early perinatal depression intervention on long-term child development outcomes: follow-up of the Thinking Healthy Programme randomised controlled trial. Lancet Psychiatry 2 (7), 609–617. 10.1016/S2215-0366(15)00109-1. 26303558

[R30] Moerbeek M, Teerenstra S, 2016. Power Analysis of Trials with Multilevel Data. CRC Press, Taylor & Francis Group.

[R31] Monahan PO, Shacham E, Reece M, Kroenke K, Ong’Or WO, Omollo O, Yebei VN, Ojwang C, 2009. Validity/reliability of PHQ-9 and PHQ-2 depression scales among adults living with HIV/AIDS in Western Kenya. J. Gen. Intern. Med 24 (2), 189–197. 10.1007/s11606-008-0846-z. 19031037 PMC2629000

[R32] Mundeva H, Snyder J, Ngilangwa DP, Kaida A, 2018. Ethics of task shifting in the health workforce: exploring the role of community health workers in HIV service delivery in low- and middle-income countries. BMC Med. Ethics 19 (71). 10.1186/s12910-018-0312-3. PMC6032788 29973217

[R33] Munodawafa M, Mall S, Lund C, Schneider M, 2018. Process evaluations of task sharing interventions for perinatal depression in low and middle income countries (LMIC): a systematic review and qualitative meta-synthesis. BMC Health Serv. Res 18 (205) 10.1186/s12913-018-3030-0. PMC5865346 29566680

[R34] Muñoz RF, Ghosh-Ippen C, Le H-N, Lieberman AF, Diaz M, La Plante L, Tandon D, Ward E, Hamil J, McGown M, Segovia M, Barkowski C, & Gier E (2001). The mothers and babies course: facilitator guide.

[R35] Okyere E, Mwanri L, Ward P, 2017. Is task-shifting a solution to the health workers’ shortage in Northern Ghana? PLoS ONE 12 (3), e0174631. 10.1371/journal.pone.0174631. 28358841 PMC5373592

[R36] Perry H, Morrow M, Borger S, , 2015. Care Groups I: An Innovative Community-Based Strategy for Improving Maternal, Neonatal, and Child Health in Resource-Constrained Settings. Glob Health Sci Pract. 3(3):358–369. doi:10.9745/GHSP-D-15-00051, 3(3), 358–369. 10.9745/GHSP-D-15-00051. 26374798 PMC4570011

[R37] Rosenbaum PR, Rubin DB, 1984. Reducing bias in observational studies using subclassification on the propensity score. J. Am. Stat. Assoc 79 (387), 516–524.

[R38] Sikander S, Lazarus A, Bangash O, Fuhr DC, Weobong B, Krishna RN, Ahmad I, Weiss HA, Price L, Rahman A, Patel V, 2015. The effectiveness and cost-effectiveness of the peer-delivered Thinking Healthy Programme for perinatal depression in Pakistan and India: the SHARE study protocol for randomised controlled trials. Trials 16 (534). 10.1186/s13063-015-1063-9. PMC4659202 26604001

[R39] Singla DR, Kumbakumba E, Aboud FE, 2015. Effects of a parenting intervention to address maternal psychological wellbeing and child development and growth in rural Uganda: a community-based, cluster-randomised trial. Lancet Global Health 3, e458–e469. 10.1016/S2214-109X(15)00099-6. 26144389

[R40] Squires J, Bricker D, Twombly E, 2015. Ages & Stages Questionnaires®: Social-Emotional, Second Edition (ASQ®:SE-2): a Parent-Completed Child Monitoring System for Social-Emotional Behaviors. Paul H. Brookes Publishing Co., Inc.

[R41] Starmann E, Collumbien M, Kyegombe N, Devries K, Michau L, Musuya T, Watts C, Heise L, 2017. Exploring couples’ processes of change in the context of SASA!, a violence against women and HIV prevention intervention in Uganda. Prevent. Sci 18 (2), 233–244. 10.1007/s11121-016-0716-6. PMC5243896 27682273

[R42] Tokhi M, Comrie-Thomson L, Davis J, Portela A, Chersich M, Luchters S, 2018. Involving men to improve maternal and newborn health: a systematic review of the effectiveness of interventions. PLoS ONE 13 (1), e0191620. 10.1371/journal.pone.0191620. 29370258 PMC5784936

[R43] Tomlinson M, Hunt X, Watt K, Naicker S, Richter L, 2020. Programmatic guidance for interventions to improve early childhood development in high HIV burden countries: a narrative review. Vulnerable Child Youth Stud. 10.1080/17450128.2020.1786204.

[R44] Turner EL, Sikander S, Bangash O, Zaidi A, Bates L, Gallis J, Ganga N, O’Donnell K, Rahman A, Maselko J, 2016. The effectiveness of the peer-delivered Thinking Healthy PLUS (THPP+) Programme for maternal depression and child socio-emotional development in Pakistan: study protocol for a three-year cluster randomized controlled trial. Trials 17 (442). 10.1186/s13063-016-1530-y. PMC5017048 27608926

[R45] UNICEF, & Countdown to 2030: Women’s, C. & A. H. (2019). Country profiles for early childhood development.

[R46] Vanobberghen F, Weiss HA, Fuhr DC, Sikander S, Afonso E, Ahmad I, Atif N, Bibi A, Bibi T, Bilal S, De Sa A, D’Souza E, Joshi A, Korgaonkar P, Krishna R, Lazarus A, Liaqat R, Sharif M, Weobong B, … Rahman A, 2020. Effectiveness of the Thinking Healthy Programme for perinatal depression delivered through peers: pooled analysis of two randomized controlled trials in India and Pakistan. J. Affect. Disord 265, 660–668. 10.1016/j.jad.2019.11.110. 32090783 PMC7042347

[R47] von Elm E, Altman DG, Egger M, Pocock SJ, Gøtzsche PC, Vandenbroucke JP, 2007. The strengthening the reporting of observational studies in epidemiology (STROBE) statement: guidelines for reporting observational studies. Bull. World Health Organ 85 (11) 10.2471/BLT.07.045120. PMC2636253 18038077

[R48] Wachs TD, Black MM, Engle PL, 2009. Maternal depression: a global threat to children’s health, development, and behavior and to human rights. Child Dev. Perspect 3 (1), 51–59. 10.1111/j.1750-8606.2008.00077.x https://doi.org/https://doi.org/.

[R49] Wainberg ML, Scorza P, Shultz JM, Helpman L, Mootz JJ, Johnson KA, Neria Y, Bradford J-ME, Oquendo MA, Arbuckle MR, 2017. Challenges and opportunities in global mental health: a research-to-practice perspective. Curr. Psychiatry Rep 19 (Issue 5) 10.1007/s11920-017-0780-z. Current Medicine Group LLC 1. PMC5553319 28425023

[R50] Wang JS-H, Ssewamala FM, Han C-K, 2014. Family economic strengthening and mental health functioning of caregivers for AIDS-affected children in rural Uganda. Vulnerable Child Youth Stud 9 (3), 258–269. 10.1080/17450128.2014.920119. 26246846 PMC4523299

[R51] Wasserstein RL, Lazar NA, 2016. The ASA statement on p-values: context, process, and purpose. Am. Stat 70 (2), 129–133. 10.1080/00031305.2016.1154108. DOI.

[R52] Worku BN, Abessa TG, Wondafrash M, Vanvuchelen M, Bruckers L, Kolsteren P, Granitzer M, 2018. The relationship of undernutrition/psychosocial factors and developmental outcomes of children in extreme poverty in Ethiopia. BMC Pediatr. 18 (45) 10.1186/s12887-018-1009-y. PMC5809114 29426302

[R53] World Health Organization. (2014). Social determinants of mental health.

[R54] World Health Organization. (2020). Improving early childhood development: WHO guideline. https://doi.org/CC BY-NC-SA 3.0 IGO. 32200595

[R55] World Health Organization, 2020b. Maternal Mental Health. World Health Organization https://www.who.int/mental_health/maternal-child/maternal_mental_health/en/.

[R56] World Health Organization, United Nations Children’s Fund, & World Bank Group. (2018). Nurturing care for early childhood development: a framework for helping children survive and thrive to transform health and human potential. https://doi.org/Licence:CC BY-NC-SA 3.0 IGO.

[R57] Yargawa J, Leonardi-Bee J, 2015. Male involvement and maternal health outcomes: systematic review and meta-analysis. J. Epidemiol. Community Health 69, 604–612. 10.1136/jech-2014-204784. 25700533 PMC4453485

