# Supplementary material for: Effect of a lay counselor delivered integrated maternal mental health and early childhood development group-based intervention in Siaya County, Kenya: A quasi-experimental longitudinal study
Source: J Affect Disord. Author manuscript; Available in PMC 2022 Feb 22. (PMC8863180; doi:10.1016/j.jad.2021.06.002)
Supplement: 1 [file NIHMS1775876-supplement-1.docx]

**Figure S1. Covariate balance with and without propensity score weighting: treated vs control**

SRQ-20 Score

Social Support-Female Friends: Yes

Social Support-Female Relatives: NA

Social Support-Female Relatives: Yes

Social Support-Husband: NA

Social Support-Husband : No Partner

Social Support-Husband: Yes

Social Support-Husband: No

Herth Hope Index

Emotional Violence: NA

Emotional Violence: Yes

Emotional Violence: No

Controlling Behavior: NA

Controlling Behavior: Yes

Controlling Behavior: No

Physical/Sexual Violence: NA

Physical/Sexual Violence: No Partner

Physical/Sexual Violence: Yes

Physical/Sexual Violence: No

Household Hunger: Severe hunger

Household Hunger: moderate

Household Hunger: little to none

Pregnancies: 4 or more pregnancies

Pregnancies: 2-3 pregnancies

Pregnancies: One pregnancy

Child General Health: Very Good

Child General Health: Good

Child General Health: Fair/Poor

Women General Health: Very Good

Women General Health: Good

Women General Health: Fair/Poor

Birth Weight: NA

Birth Weight: Still Pregnant

Birth Weight: Low Birth Weight

Birth Weight: Normal Weight

Work in last 7 days: No

Household Size

Age in Years

Early Stimulation Behaviors Total Score

0.0

0.1

0.2

0.3

0.4

Absolute Mean Differences

Unadjusted

IPW

OW

Covariate Balance

Unadjusted, the unadjusted study population without propensity score weighting; IPW, the study population weighted by inverse probability weights; OW, the study population weighted by overlap weights; higher absolute mean difference represents larger covariate imbalance between the intervention and control arms

**Figure S2. Covariate balance with and without propensity score weighting: high attendance vs low attendance**

0.00

0.05

0.10

0.15

Absolute Mean Differences

Unadjusted

IPW

OW

Covariate Balance

Early Stimulation Behaviors Total Score

Age in Years

Household Size

Work in last 7 days: No

Birth Weight: Normal Weight

Birth Weight: Low Birth Weight

Birth Weight: Still Pregnant

Women General Health: Fair/Poor

Women General Health: Good

Women General Health: Very Good

Child General Health: Fair/Poor

Child General Health: Good

Child General Health: Very Good

Pregnancies: One pregnancy

Pregnancies: 2-3 pregnancies

Pregnancies: 4 or more pregnancies

Household Hunger: little to none

Household Hunger: moderate

Household Hunger: Severe hunger

Physical/Sexual Violence: No

Physical/Sexual Violence: Yes

Physical/Sexual Violence: No Partner

Physical/Sexual Violence: NA

Controlling Behavior: No

Controlling Behavior: Yes

Controlling Behavior: NA

Emotional Violence: No

Emotional Violence: Yes

Herth Hope Index

Social Support-Husband: No

Social Support-Husband: Yes

Social Support-Husband : No Partner

Social Support-Husband: NA

Social Support-Female Relatives: Yes

Social Support-Female Relatives: NA

Social Support-Female Friends: Yes

SRQ-20 Score

Unadjusted, the unadjusted study population without propensity score weighting; IPW, the study population weighted by inverse probability weights; OW, the study population weighted by overlap weights; higher absolute mean difference represents larger covariate imbalance between the high and low attendance groups

**Figure S3. Margin plot for PHQ-9 score**

**Figure S4. Margin plot for mean ASQ-SE score**

**Figure S5. Exploratory analysis (high attendance vs low attendance): margin plot for PHQ-9 score**

**Figure S6. Exploratory analysis (high attendance vs low attendance): margin plot for mean ASQ-SE score**

**Figure S7. Effect modification on PHQ-9 difference in differences at follow-up 1**

Abbreviations: ASQ-SE, ages and stages questionnaire-social emotional score; SS, social support; IPV, intimate partner violence; PHQ-9, patient health questionnaire score.

**Figure S8. Effect modification on PHQ-9 difference in differences at follow-up 2**

Abbreviations: ASQ-SE, ages and stages questionnaire-social emotional score; SS, social support; IPV, intimate partner violence; PHQ-9, patient health questionnaire score.

**Figure S9. Effect modification on PHQ-9 difference in differences at follow-up 3**

Abbreviations: ASQ-SE, ages and stages questionnaire-social emotional score; SS, social support; IPV, intimate partner violence; PHQ-9, patient health questionnaire score.

**Figure S10. Effect modification on ASQ Difference at follow-up 1**

Abbreviations: ASQ-SE, ages and stages questionnaire-social emotional score; SS, social support; IPV, intimate partner violence; PHQ-9, patient health questionnaire score.

**Figure S11. Effect modification on ASQ Difference at follow-up 2**

Abbreviations: ASQ-SE, ages and stages questionnaire-social emotional score; SS, social support; IPV, intimate partner violence; PHQ-9, patient health questionnaire score.

**Figure S12. Effect modification on ASQ Difference at follow-up 3**

Abbreviations: ASQ-SE, ages and stages questionnaire-social emotional score; SS, social support; IPV, intimate partner violence; PHQ-9, patient health questionnaire score.
